# Properties of Beef Patties with *Tenebrio molitor* Powder as a Meat Replacer During Storage

**DOI:** 10.3390/foods14213707

**Published:** 2025-10-30

**Authors:** Camila Cristina A. de Sousa, Rafael Sepúlveda F. Trevisan Passos, Claudia Ruiz-Capillas, Ana M. Herrero, Maurício Costa A. da Silva, Carlos Pasqualin Cavalheiro

**Affiliations:** 1 Laboratório de Indústria e Inspeção de Carnes e Derivados, Universidade Federal da Bahia, Salvador 40170-115, BA, Brazil; camila.cristina@ufba.br (C.C.A.d.S.); rafael.sepulveda@ufba.br (R.S.F.T.P.); mcasilva@ufba.br (M.C.A.d.S.); 2 INDMEAT Group, Department of Meat and Fish Products, Institute of Food Science, Technology and Nutrition (ICTAN-CSIC), 28040 Madrid, Spain; ana.herrero@ictan.csic.es

**Keywords:** yellow mealworm, novel ingredients, reformulation, meat products, entomophagy

## Abstract

This study aimed to assess the effects of partially replacing lean beef with *Tenebrio molitor* powder at different levels on the physicochemical and microbiological properties of beef patties throughout refrigerated storage. Four treatments were prepared: Control (beef), HT50 (5% *T. molitor* powder), HT75 (7.5% *T. molitor* powder), and HT100 (10% *T. molitor* powder). The inclusion of *T. molitor* powder significantly increased (*p* < 0.05) the protein content while reducing cooking loss and diameter reduction (*p* < 0.05). Color analyses revealed a darker appearance in reformulated patties throughout storage (L* = 36.8–41.2; a* = 6.3–9.3; ΔE* = 4.7–10.4), particularly in HT75 and HT100, compared with control (L* = 43.4–45.5; a* = 10.0–13.7). Kramer shear force values remained comparable (*p* > 0.05) to the control in HT50 but increased (*p* < 0.05) in HT75 and HT100 during storage. Lipid oxidation increased (*p* < 0.05) over time in reformulated treatments; however, initial TBARS values were lower than those of the control. Microbiological analysis showed significantly reduced (*p* < 0.05) mesophilic and *Enterobacteriaceae* counts in *T. molitor* powder formulations. The presence of *Bacillus cereus* was attributed to *T. molitor* powder, as no growth was detected in the control. Among reformulated treatments, HT50 proved to be the most suitable beef patty formulation, as it maintained key physicochemical attributes such as color stability, texture, and oxidative stability, while providing nutritional benefits.

## 1. Introduction

Rising global meat demand has intensified environmental concerns, particularly greenhouse gas emissions, deforestation, and water depletion associated with livestock production [1]. These pressures have heightened consumer awareness and stimulated the food industry to explore sustainable protein alternatives, such as algae, mushrooms [2], cultured meat [3], plant-based proteins [4], and edible insects [5].

Edible insects have gained attention for their substantially lower land, water, and feed requirements than conventional livestock [6]. In particular, *Tenebrio molitor* larvae (yellow mealworms) are notable for their high protein content, low chitin levels that enhance protein bioavailability and digestibility [7,8], and a rich profile of essential amino acids, fatty acids, and micronutrients [9]. Reflecting this potential, the European Food Safety Authority has approved the commercialization of *T. molitor* larvae in frozen, dried, and powdered forms [10].

Van Huis et al. [11] highlight that edible insects represent a promising strategy to combat malnutrition and ensure food security, particularly in countries with limited access to food, especially animal-derived proteins. However, despite their high nutritional value, the authors emphasize that edible insects should be considered complementary protein sources rather than complete substitutes for meat or other animal-based proteins. Due to their amino acid composition, Oliveira et al. [8] noted that edible insects should be included in the diet with different protein sources, particularly of animal origin. Therefore, edible insects should be incorporated into the human diet to complement and enhance overall protein quality while promoting sustainable nutrition and greater dietary diversity.

Despite these nutritional and environmental advantages, consumer acceptance of edible insects in Western diets remains limited by cultural barriers and pronounced food neophobia [12]. Incorporating insect powder into familiar food matrices, such as meat products, has emerged as a promising strategy to improve acceptance [13]. Reformulated meat products partially replace conventional meat with insect proteins and provide environmental benefits while maintaining desirable sensory and nutritional qualities [14].

Developing reformulated meat products with satisfactory physicochemical, technological, and sensory attributes remains challenging, especially regarding texture, oxidative stability, and microbial safety during storage [15,16]. Patties represent an ideal platform for integrating edible insect powders, providing a practical approach to promote entomophagy within a familiar format [17]. Although some studies have evaluated *Hermetia illucens* [18], *Gryllus assimilis* [19], and *Acheta domesticus* [20] powders in patty formulations, data on beef patties enriched with *T. molitor* powder are scarce. Therefore, this study aimed to assess the effects of partially replacing lean beef with *T. molitor* powder at inclusion levels of 5.0%, 7.5%, and 10.0% on the physicochemical and microbiological characteristics of beef patties during refrigerated storage.

## 2. Materials and Methods

### 2.1. Raw Materials

The *T. molitor* powder (50.0% protein, 30.8% fat, 6.7% carbohydrates, 3.3% fiber) was sourced from Nimavert (Meise, Belgium). Fresh lean beef (75.65% moisture, 21.69% protein, 3.32% fat, and 1.02% ash) was obtained from a local slaughterhouse in Madrid, Spain. The meat was ground using a 6 mm plate meat grinder and frozen for 7 days until use. Ice/water, sodium chloride (Panreac Química S.A., Barcelona, Spain), and onion powder (Pilarica, S.A., Valencia, Spain) completed the ingredient list for patty formulation.

### 2.2. Beef Patties Manufacturing

**Figure 1:**
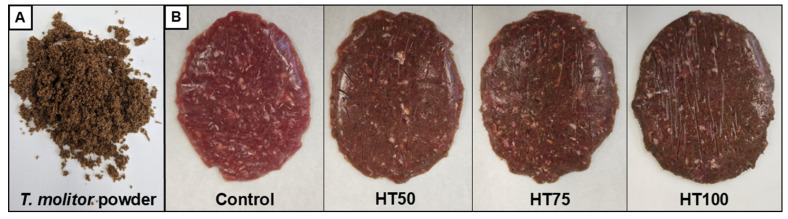
Typical appearance of *T. molitor* powder (**A**) and raw patties elaborated with different levels of *T. molitor* powder (**B**). Treatments: Control: patties without *T. molitor* powder addition; HT50: patties with 5% *T. molitor* powder; HT75: patties with 7.5% *T. molitor* powder; HT100: patties with 10% *T. molitor* powder.

The control formulation comprised 86.5% lean beef meat, 10.0% ice/waterr, 2.0% onion powder, and 1.5% sodium chloride. Three reformulated treatments were prepared by replacing lean beef meat with *T. molitor* powder (Figure 1A) at inclusion levels of 5.0% (HT50), 7.5% (HT75), and 10.0% (HT100). For each batch, lean beef and half of the ingredients were homogenized in a Hobart (Model N50-6, Troy, OH, USA) for 1 min. The remaining ingredients were added and mixed for 1 min, followed by a final 1 min homogenization to ensure a complete mixture. Throughout processing, the temperature was maintained at 10 °C. The resulting patty mixture was divided into 90 g portions, shaped in a patty mold, packaged in polyethylene bags, and stored at 4 °C for analysis. The visual appearance of raw patties is shown in Figure 1B.

### 2.3. Proximate Composition

Moisture (950.46) and ash (920.153) contents of raw patties were determined in triplicate following AOAC [21] standard procedures. Protein content was quantified using a LECO FP-200 Nitrogen Determinator (Leco Corp., St. Joseph, MI, USA). Total lipids were extracted according to the procedure of Bligh and Dyer [22].

### 2.4. Cooking Loss and Diameter Reduction

Patties were grilled on an electric grill (JATA GR3000, Navarra, Spain) until an internal temperature of 72 °C. After grilling, samples were cooled to 25 °C. Measurements were performed in triplicate for each treatment, and the results were expressed as a percentage and calculated using the formula [23]:

$$CL\left(\%\right)=\frac{{Weight}_{raw}-{Weight}_{grilled}}{{Weight}_{raw}}\times 100$$

Diameter reduction (DR) was evaluated by measuring the difference between the diameter before and after the grilling process using a ruler, and calculated as [23]:

$$DR\left(\%\right)=\frac{{Diameter}_{raw}-{Diameter}_{grilled}}{{Diameter}_{raw}}\times 100$$

### 2.5. pH

Ten grams of each raw patty sample were homogenized with 90 mL of distilled water, and the pH was recorded in triplicate at 25 °C using a digital pH meter (model 827, pH LabMethrom, Herisau, Switzerland) previously calibrated with standard buffer solutions of pH 4.00 and 7.00 [21].

### 2.6. Instrumental Color

Surface color of raw patties was assessed following AMSA [24] guidelines. After a 10 min exposure to air, five measurements per sample were taken with a portable colorimeter (Konica Minolta CR-400, Tokyo, Japan), previously calibrated with the white reference plate according to the manufacturer’s instructions, under a D-65 illuminant and 10° standard observer with an 8 mm aperture. Lightness (L*), redness (a*), and yellowness (b*) were measured. Additionally, the total color difference (ΔE*) relative to the control was calculated as:

(1) $${∆E}^{*}=\;\sqrt{(L-L_{0})²+\;\left(a-a_{0}\right)²+(b-b_{0})²}$$

### 2.7. Kramer Shear Force

Grilled patties were cut into 2 × 2 cm samples, weighed, and tested at room temperature in a TA-XTplus Texture Analyzer (Stable Micro Systems Ltd., Godalming, UK) equipped with a miniature Kramer (HDP/MK05) 5-bladed cell and a 25 kg load cell. Samples were sheared to a 20 mm compression at 0.8 mm/s crosshead speed. Five measurements per treatment were taken, and results were expressed as the maximum force per gram of sample (N/g) [20].

### 2.8. Lipid Oxidation

Thiobarbituric acid reactive substances (TBARS) were determined according to Triki et al. [25]. Five grams of raw patties were homogenized with 35 mL of 7.5% trichloroacetic acid in an Ultraturrax blender (Ika-Werke, GmbH & Co., Staufen, Germany) for 1 min. The homogenate was centrifuged at 3000× *g* for 2 min, and the supernatant was filtered. Five milliliters of filtrate were reacted with 5 mL of 20 mM thiobarbituric acid, incubated in the dark at 20 °C for 20 h, and the absorbance at 532 nm was measured (Lambda 15 UV/VIS spectrophotometer, Perkin-Elmer, Springfield, IL, USA). Malondialdehyde (MDA) concentration was calculated against a 1,1,3,3-tetraethoxypropane (Sigma Chemical Co., St. Louis, MO, USA) calibration curve and expressed as mg MDA/kg of sample.

### 2.9. Microbiological Analysis

Ten grams of each raw patty treatment were homogenized with 90 mL of buffered peptone water (Panreac, Darmstadt, Germany). Mesophilic aerobic counts were performed on Plate Count agar (37 °C, 48 h), and lactic acid bacteria (LAB) on de Man, Rogosa, and Sharpe agar (37 °C, 48 h). *Enterobacteriaceae* were enumerated on Violet Red Bile Glucose agar (37 °C, 24 h), and *Bacillus cereus* on Mannitol Egg Yolk Polymyxin agar (30 °C, 24 h), with confirmatory tests. Additionally, the presence of *Salmonella* spp. and *Listeria monocytogenes* was determined according to APHA [26] methodology. All microbiological analyses were performed in duplicate, and results were expressed as log CFU/g.

### 2.10. Statistical Analysis

Data were analyzed by two-way ANOVA using SPSS 25.0 (SPSS Inc., Chicago, IL, USA) to analyze the effects of the storage and the different concentrations of *T. molitor* powder as a meat replacer on the physicochemical and microbiological properties of beef patties. A completely randomized design included treatment groups (control, HT50, HT75, and HT100) and storage times (days 1, 3, and 6) as fixed effects and two replicates as a random effect. Means were compared using Tukey, and differences were considered significant when *p <* 0.05.

## 3. Results

### 3.1. Proximate Composition

The proximate composition of reformulated beef patties is presented in Table 1. Moisture content ranged from 66.22 to 72.46 g/100 g, with the control exhibiting the highest value (*p* < 0.05). Moisture content decreased progressively as the inclusion level of *T. molitor* powder increased. Conversely, protein content rose significantly (*p* < 0.05) with *T. molitor* powder addition, peaking in the HT100 treatment at 22.99 g/100 g. Although HT50 and HT75 did not differ from each other (21.51 and 21.92 g/100 g, respectively), all reformulated patties showed higher protein content than the control (19.31 g/100 g). Fat levels remained constant across treatments, ranging from 5.65 to 6.63 g/100 g. Ash content increased (*p* < 0.05) in formulations with higher *T. molitor* powder levels (2.48–2.71 g/100 g) compared with the control (2.36 g/100 g).

### 3.2. Cooking Loss and Diameter Reduction

**Figure 2:**
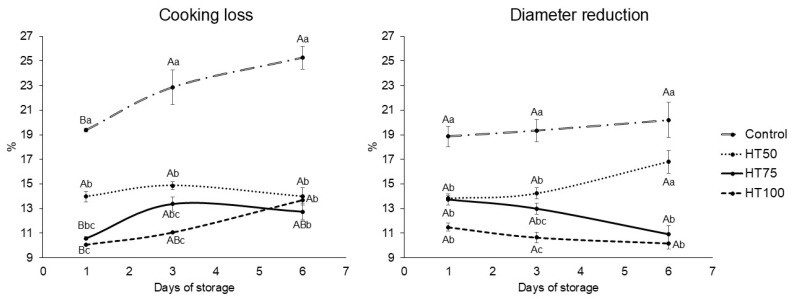
Cooking loss (%) and Diameter reduction (%) of patties with *T. molitor* powder addition. Different uppercase superscripts (A,B) indicate significant differences (*p* ≤ 0.05) among the same treatment over storage days. Different lowercase superscripts (a–c) indicate significant differences (*p* ≤ 0.05) among different treatments on the same day. Treatments: Control: patties without *T. molitor* powder addition; HT50: patties with 5% *T. molitor* powder; HT75: patties with 7.5% *T. molitor* powder; HT100: patties with 10% *T. molitor* powder.

Incorporating *T. molitor* powder reduced CL in all reformulated treatments compared to the control throughout storage (*p* < 0.05; Figure 2). On day 1, CL ranged from 10.05 to 19.36%, and significant differences were found between HT50 (13.97%) and HT100 (10.05%). Conversely, no differences were observed between HT50 and HT75 (10.58%) or between HT75 and HT100. A comparable trend persisted on day 3, with CL values ranging from 11.05% to 22.85%. By day 6, the control (25.26%) had the highest CL (*p* < 0.05), whereas CL values among the reformulated patties were similar (*p* > 0.05), ranging from 12.74 to 13.98%. Except for HT50, CL increased over storage (*p* < 0.05).

The DR remained unaffected by storage time (*p* > 0.05, Figure 2). Nevertheless, the reformulated beef patties exhibited significantly lower (*p* < 0.05) DR values than the control on days 1 and 3. On day 1, the DR value of the control was 18.86%, whereas those of the reformulated treatments ranged from 11.49 to 13.85%. On day 3, the control exhibited a DR value of 19.34%, while HT75 (12.99%) showed statistical similarity with HT50 (14.25%) and HT100 (10.64%), which differed from each other (*p* < 0.05). At the end of the storage, HT50 (16.79%) showed no significant difference (*p* > 0.05) compared to the control (20.20%), whereas HT75 and HT100 retained lower DR values (*p* < 0.05), of 10.91 and 10.15%, respectively.

### 3.3. pH

**Figure 3:**
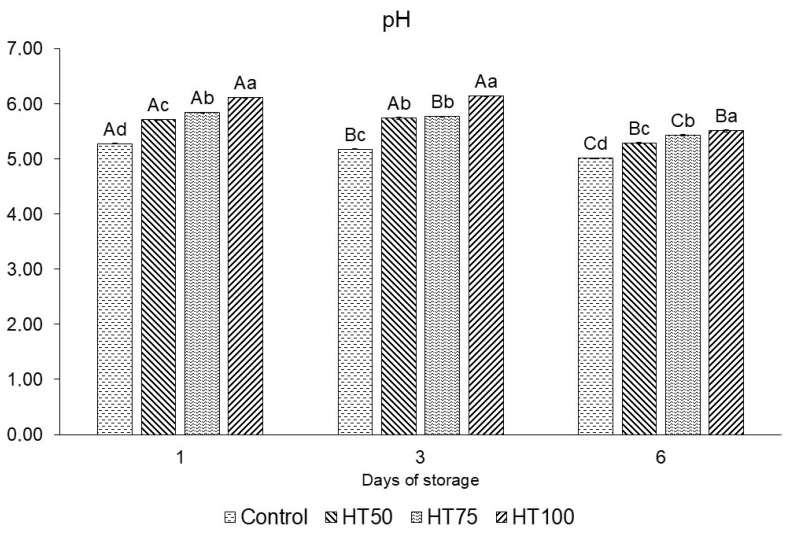
pH of raw patties with *T. molitor* powder addition during storage. Different uppercase superscripts (A–C) indicate significant differences (*p* ≤ 0.05) among the same treatment over storage days. Different lowercase superscripts (a–d) indicate significant differences (*p* ≤ 0.05) among different treatments on the same day. Treatments: Control: patties without *T. molitor* powder addition; HT50: patties with 5% *T. molitor* powder; HT75: patties with 7.5% *T. molitor* powder; HT100: patties with 10% *T. molitor* powder.

On day 1, the pH values were consistently higher (*p* < 0.05) in treatments containing *T. molitor* powder compared with the control (5.28), increasing (*p* < 0.05) proportionally with the inclusion level, reaching 5.71, 5.84, and 6.11 in HT50, HT75, and HT100, respectively (Figure 3). Among all treatments, HT100 exhibited the highest pH values throughout the entire storage period. The pH values of HT50 (5.74) and HT100 (6.14) remained stable on day 3, whereas HT75 (5.77) and the control (5.18) showed a significant decrease (*p* < 0.05). By day 6, all samples showed a pH reduction: HT50, HT75, and HT100 recorded values of 5.29, 5.43, and 5.51, respectively, while the control reached the lowest value at 5.01 (Figure 3).

### 3.4. Instrumental Color

Overall, the L* and a* parameters were significantly affected (*p* < 0.05) by the inclusion of *T. molitor* powder (Table 2). At the beginning of the storage, lightness values decreased (*p* < 0.05) as the level of *T. molitor* powder increased, ranging from 36.8 ± 0.6 to 42.9 ± 0.5, with HT75 and HT100 presenting the lowest values. This finding indicates that reformulated beef patties became progressively darker with higher proportions of *T. molitor* powder (Figure 1B). On day 3, the control treatment exhibited the highest lightness value (43.4 ± 0.6), while no significant differences were observed among the *T. molitor* treatments (37.5 ± 0.4–38.5 ± 0.2). By the end of storage, lightness declined in reformulated beef patties as *T. molitor* powder levels increased (38.2 ± 0.3–41.1 ± 0.5) compared with the control (45.5 ± 0.6).

Similar trends were observed for a* values, with the control maintaining the highest redness throughout storage (Table 2). On day 1, redness values in reformulated treatments ranged from 6.7 ± 0.3 to 7.6 ± 0.3, whereas the control reached 10.0 ± 0.3. The a* values of the control (13.7 ± 0.4), HT50 (9.2 ± 0.4), and HT75 (8.3 ± 0.3) increased by day 3, while HT100 (6.7 ± 0.2) remained stable. By the end of the storage, a* values ranged from 6.3 ± 0.2 to 13.3 ± 0.3, directly correlating with the level of *T. molitor* powder inclusion, demonstrating that reformulated beef patties exhibited lower redness than the control.

In contrast, b* values remained unaffected by storage across all treatments. No significant differences in yellowness were detected among treatments on days 1 (8.1 ± 0.3–9.2 ± 0.3) and 6 (8.7 ± 0.2–9.6 ± 0.3). On day 3, HT50 (9.6 ± 0.3) exhibited the highest b* value compared to the other treatments (8.4 ± 0.2–8.5 ± 0.3), whose values were similar (*p* < 0.05). Analysis of total color differences revealed that both treatment and storage period significantly affected (*p* < 0.05) color variation. ΔE* values increased with higher levels of *T. molitor* powder, ranging from 4.7 ± 0.5 to 7.2 ± 0.7 on day 1. On day 3, HT50 and HT100 showed greater (*p* < 0.05) total color differences compared to day 1, reaching 6.9 ± 0.4 and 9.5 ± 0.4, respectively, whereas HT75 remained stable (8.5 ± 0.4). No significant differences were observed between the final ΔE* values of HT50 (6.2 ± 0.4) and HT75 (8.7 ± 0.4) and their initial values. Conversely, HT100 exhibited the most pronounced storage-induced change, indicating that color differences became more evident during storage of reformulated beef patties with higher *T. molitor* powder levels.

### 3.5. Kramer Shear Force

The KSF values did not differ significantly among treatments on day 1, ranging from 26.88 ± 0.51 to 30.32 ± 0.97 N/g (*p* > 0.05; Table 3). By day 6, HT75 and HT100 exhibited higher KSF values (42.45 ± 1.25 N/g and 43.34 ± 0.27 N/g, respectively; *p* < 0.05) compared to HT50 (34.92 ± 1.42 N/g) and the control (32.86 ± 0.52 N/g), which did not differ from each other. Overall, storage significantly increased KSF values in treatments containing *T. molitor* powder (*p* < 0.05). Notably, HT50 did not differ (*p* > 0.05) from the control on day 6 (Table 3).

### 3.6. Lipid Oxidation

The control treatment exhibited the highest (*p* < 0.05) TBARS value on day 1 (1.63 ± 0.02 mg MDA/kg). In contrast, reformulated patties showed progressively lower TBARS as *T. molitor* inclusion increased, ranging from 0.92 ± 0.01 to 1.32 ± 0.01 mg MDA/kg. A similar trend appeared on day 6, with the control (1.63 ± 0.01 mg MDA/kg) still surpassing all reformulated patties in lipid oxidation (*p* < 0.05). Among the reformulated beef patties, HT50 recorded the highest TBARS (*p* < 0.05) value (1.51 ± 0.01 mg MDA/kg), followed by HT100 (1.45 mg MDA/kg), which did not differ statistically from either HT50 or HT75.

Interestingly, the control treatment maintained oxidative stability over storage (*p* > 0.05), while all reformulated patties experienced significant increases between days 1 and 6 (*p* < 0.05), with magnitude proportional to *T. molitor* powder level.

### 3.7. Microbiological Analysis

**Figure 4:**
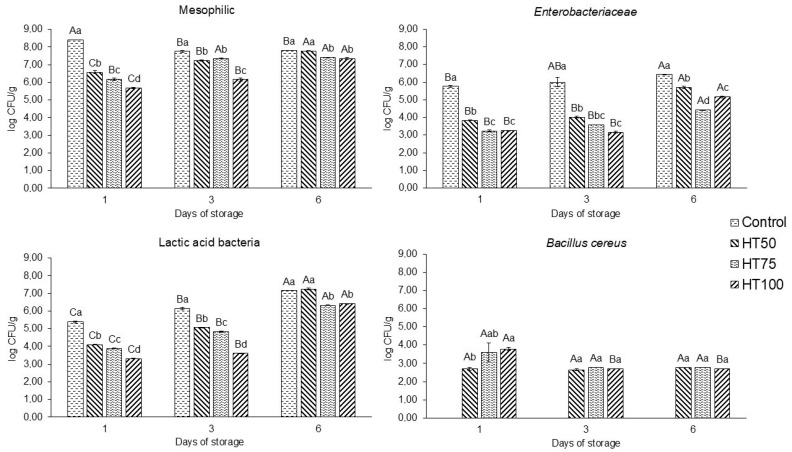
Microbiological counts (log CFU/g) of raw patties with *T. molitor* powder addition during storage. Different uppercase superscripts (A–C) indicate significant differences (*p* ≤ 0.05) among the same treatment over storage days. Different lowercase superscripts (a–d) indicate significant differences (*p* ≤ 0.05) among different treatments on the same day. Treatments: Control: patties without *T. molitor* powder addition; HT50: patties with 5% *T. molitor* powder; HT75: patties with 7.5% *T. molitor* powder; HT100: patties with 10% *T. molitor* powder.

On day 1, mesophilic counts decreased (*p* < 0.05) with rising levels of *T. molitor* powder (Figure 4). The control treatment exhibited the highest count on day 1 (8.41 log CFU/g), whereas reformulated beef patties containing higher proportions of *T. molitor* powder showed lower counts (5.67–6.17 log CFU/g; *p* < 0.05) compared to HT50 (6.58 log CFU/g). However, mesophilic counts increased in reformulated beef patties over storage, while those in the control decreased (*p* < 0.05). Nevertheless, the control count (7.75 log CFU/g) remained higher than that of the *T. molitor* treatments (6.16–7.36 log CFU/g). By day 6, HT50 and control treatments converged to similar values (*p* > 0.05), reaching 7.78 log CFU/g and 7.81 log CFU/g, respectively, whereas HT75 and HT100 exhibited lower counts (7.36–7.41 log CFU/g; *p* < 0.05).

*Enterobacteriaceae* counts in the control treatment were significantly higher (*p* < 0.05) than those in all reformulated beef patty treatments throughout storage (Figure 4). *T. molitor* powder treatments exhibited counts ranging from 3.24 to 3.84 log CFU/g, whereas the control registered 5.76 log CFU/g on day 1. On day 3, all treatments showed *Enterobacteriaceae* counts similar to their initial values, with *T. molitor* treatments ranging from 3.17 to 4.02 log CFU/g and the control reaching 6.00 log CFU/g. In contrast, reformulated treatments exhibited a notable increase in *Enterobacteriaceae* counts by the end of the storage period (*p* < 0.05), reaching values between 4.42 and 5.70 log CFU/g. At the same time, the control remained stable at 6.43 log CFU/g.

Lactic acid bacteria (LAB) counts decreased as the levels of *T. molitor* powder increased, although all treatments showed a significant rise in LAB over time (*p* < 0.05). On day 1, LAB counts reached 5.39 log CFU/g in the control and ranged from 3.32 to 4.10 log CFU/g among reformulated treatments, showing a progressive reduction inversely proportional to the amount of *T. molitor* powder added. A similar trend was observed on day 3, with counts ranging from 3.61 to 5.06 log CFU/g in *T. molitor* treatments and 6.14 log CFU/g in the control. By the end of storage, HT50 exhibited the highest LAB count among the reformulated treatments (6.33–7.25 log CFU/g), comparable (*p* < 0.05) to that of the control (7.15 log CFU/g). Overall, higher proportions of *T. molitor* powder resulted in lower microbial counts.

*B. cereus* emerged as an important species in reformulated beef patties. According to Figure 4, no growth of *B. cereus* was observed in the control treatment. *B. cereus* counts ranged from 2.69 to 3.78 CFU/g at the beginning of storage. Remarkably, these counts remained relatively stable during the storage period, varying between 2.65 and 2.78 log CFU/g on day 3 and 2.70 and 2.78 log CFU/g on day 6. Importantly, neither *Salmonella* spp. nor *L. monocytogenes* were detected in any patty throughout the storage period.

## 4. Discussion

### 4.1. Proximate Composition

Moisture, protein, and ash changes directly reflect the powder’s proximate composition. The lower moisture and higher protein levels associated with increasing proportions of *T. molitor* powder can be explained by its high total solids content (93–95%) [27], compared with the beef used in patty manufacturing. The moisture content of *T. molitor* powder (5.41%) [28] is considerably lower than that of beef (75.65%), contributing to the reduced moisture levels in the reformulated beef patties. Likewise, the progressive increase in protein content with higher *T. molitor* inclusion levels was expected, given its higher protein concentration (50%) than beef (21.69%). The higher protein content in insect-based reformulated meat products contributes to a more compact protein matrix formation, restricting water mobility and lowering moisture levels [16]. Previous studies have also reported increased protein content in meat products with *T. molitor* powder, such as frankfurters [27,29] and pork patties [30]. Despite the high lipid content of *T. molitor* powder (30.8%), the fat content of the reformulated beef patties remained stable, consistent with findings from frankfurters reformulated with insect powder [29] and aligns with nutritional goals to limit excessive fat intake [31]. The increase in ash content in formulations with higher *T. molitor* powder levels likely reflects residual chitin from the insect powder [1].

### 4.2. Cooking Loss and Diameter Reduction

Cooking loss (CL) and diameter reduction (DR) are key indicators of protein functionality during cooking [20,32]. The increase in CL over storage reflects progressive protein denaturation [33]. The reduced CL observed in reformulated beef patties likely arises from insect powder’s enhanced water and oil-holding capacities, which are positively correlated with the substitution level [20]. Our findings are consistent with those reported by Gomes Martins et al. [19], who observed higher cooking yields in *G. assimilis*-enriched patties, and other studies on reformulated meat products [1,27,28].

Similarly to CL, the reduced DR values can also be attributed to the high solubility and strong binding capacity of *T. molitor* powder proteins with meat protein [19]. As previously mentioned, this effect is associated with the increased protein content, which promotes the formation of a denser structure after cooking, improving water and fat retention within the matrix and thereby reducing exudation [16]. According to Belucci et al. [34], there is a direct relationship between DR and CL, as the reduction in diameter of beef patties results from protein denaturation during heat treatment, which increases water and fat losses.

### 4.3. pH

Higher pH values in treatments containing *T. molitor* powder were expected, given the naturally higher pH of *T. molitor* powder, which ranges from 6.32 to 7.00 [1,28], showing a more alkaline character than that of the lean beef used (5.61). The overall decline in pH during storage may be attributed to the onset of spoilage processes and the growth of lactic acid bacteria (LAB), whose metabolic activity produces organic acids that reduce pH values. Our findings are consistent with previous studies on pork patties and emulsified meat products enriched with *T. molitor* powder [1,28,30], which reported higher pH values than their control samples.

### 4.4. Instrumental Color

Color is a critical quality attribute in reformulated beef patties, strongly influencing consumers’ initial perception. As shown in Figure 1, reformulated beef patties were expected to become progressively darker (Figure 1B) with increasing levels of *T. molitor* powder. This effect is associated with melanin, which imparts the characteristic dark pigmentation of the powder (Figure 1A), leading to a darker, less red, and more brownish appearance, reflected by the reduction in L* and a* values. Previous studies also reported similar darkening effects in reformulated beef patties with edible insect powders [18,19,20]. L* values in meat products are influenced by pH, moisture content, and water mobility. The higher alkalinity observed in reformulated beef patties results in reduced light scattering and a darker appearance. In contrast, the lower pH in the control is associated with greater light reflectance, lower water-holding capacity, and a lighter color [35].

Changes in a* values can be mainly attributed to the conversion of carboxymyoglobin into deoxymyoglobin and metmyoglobin, which differ in stability and may exhibit greater resistance to typical color changes in meat products during storage [36]. This may explain the increase in redness in the control and HT50 samples over storage, where myoglobin pigments exert a more decisive influence than in HT75 and HT100, which displayed greater color stability due to higher *T. molitor* powder content.

Regarding total color differences, except for HT50 on day 1, all ΔE* values exceeded the perceptibility threshold of 5.0 (Table 2), confirming visually noticeable color differences between reformulated beef patties and the control [37].

### 4.5. Kramer Shear Force

Similar increases in KSF values have been reported for patties fortified with edible insect powders [20,30]. This phenomenon is often attributed to the reduced moisture content and higher solid fraction in meat products containing insect powder, a combination associated with forming a denser and more cohesive meat matrix, resulting in a firmer texture [16,27]. As Kim et al. [38] demonstrated, *T. molitor* powder has a limited capacity to establish protein–protein networks, which may impair water retention and promote moisture loss, thereby increasing shear resistance.

Notably, HT50 did not differ (*p* > 0.05) from the control on day 6, indicating that a 5.0% inclusion of *T. molitor* powder can be incorporated into reformulated beef patties without compromising textural quality.

### 4.6. Lipid Oxidation

The findings of this study align with those of Choi et al. [30], who reported similar TBARS rises in reformulated pork patties with insect powder addition after 7 days of storage.

Although fat content was uniform across treatments, *T. molitor* powder contains about 30.8% lipids, mostly polyunsaturated fatty acids prone to oxidation. Oxidative stability in reformulated meat products may depend on powder composition, insect slaughtering methods, and drying techniques [39].

Except for HT100 on day 1 (Table 3), all treatments exceed the 1.0 mg MDA/kg TBARS threshold associated with rancid odors in meat products [40]. In this regard, a promising approach would be incorporating natural antioxidants capable of inhibiting lipid oxidation, thereby preserving the oxidative stability and extending the shelf life of beef patties [41].

### 4.7. Microbiological Analysis

Mesophilic aerobic bacteria, *Enterobacteriaceae*, lactic acid bacteria (LAB), and *Bacillus cereus* are among the predominant microbial groups naturally present in edible insects, emphasizing the importance of establishing specific regulatory frameworks to ensure microbiological safety of insect-based food products [15]. Despite this, the initial mesophilic counts were within the typical range reported for raw patties (6.32 to 6.86 log CFU/g) [41,42]. Therefore, cooking to a minimum internal temperature of 72 °C is recommended to ensure consumer safety.

The increase in LAB counts during storage aligns with previous findings for beef patties [42], and the values observed were consistent with those reported by Cavalheiro et al. [20] for similar reformulated beef patties. Overall, higher proportions of *T. molitor* powder led to lower microbial counts, suggesting an inherent antimicrobial effect. Bioactive peptides derived from edible insects may inhibit spoilage and pathogenic microorganisms, potentially extending the shelf life of reformulated meat products [43].

The growth of *B. cereus* in insect powders is a concern due to its ability to form heat- and desiccation-resistant endospores [15]. Although *B. cereus* counts remained stable throughout storage, its occurrence, likely introduced via soil or insect gut microbiota, underscores the necessity of stringent processing and decontamination steps for insect powder to safeguard microbial quality and public health [44]. Importantly, neither *Salmonella* spp. nor *L. monocytogenes* were detected in any patty throughout the storage period.

## 5. Conclusions

Incorporating *T. molitor* powder as a partial meat replacer in beef patties significantly enhanced protein content while reducing cooking losses and diameter shrinkage. Although higher *T. molitor* powder levels produced darker color, increased KSF values, and greater lipid oxidation over time, HT50 achieved the best overall balance. It proved to be the most suitable beef patty formulation, maintaining key physicochemical attributes such as color stability, texture, and oxidative stability, while providing nutritional benefits. By addressing quality changes over time, these results support the development of more stable, sustainable reformulated meat products using edible insect proteins. Future studies should elucidate the mechanisms driving microbial growth in these reformulated beef patties and evaluate the incorporation of suitable preservatives to improve microbiological and oxidative stability during refrigerated storage.

## Figures and Tables

**Table 1:** Proximate composition (g/100 g) of patties with different levels of *T. molitor* powder.

Parameter	Treatment			
	Control	HT50	HT75	HT100
Protein	19.31 ± 0.12 ^c^	21.51 ± 0.17 ^b^	21.92 ± 0.15 ^b^	22.99 ± 0.12 ^a^
Moisture	72.46 ± 0.18 ^a^	68.69 ± 0.11 ^b^	68.44 ± 0.30 ^b^	66.22 ± 0.19 ^c^
Fat	5.65 ± 0.14 ^a^	6.01 ± 0.10 ^a^	6.51 ± 0.13 ^a^	6.63 ± 0.51 ^a^
Ash	2.36 ± 0.03 ^c^	2.48 ± 0.03 ^bc^	2.56 ± 0.01 ^ab^	2.71 ± 0.06 ^a^

Means ± SEM. Means with different superscripts (^a–c^) in the same row indicate significant differences. SEM: Standard error of the mean. Control: patties without *T. molitor* powder addition; HT50: patties with 5% *T. molitor* powder; HT75: patties with 7.5% *T. molitor* powder; HT100: patties with 10% *T. molitor* powder.

**Table 2:** Color parameters of raw patties with different levels of *T. molitor* powder during storage.

Parameter	Day	Treatments			
		Control	HT50	HT75	HT100
L*	1	42.9 ± 0.5 ^Ba^	41.2 ± 0.6 ^Aa^	36.9 ± 0.5 ^Bb^	36.8 ± 0.6 ^Ab^
	3	43.4 ± 0.6 ^Aba^	38.5 ± 0.2 ^Bb^	37.9 ± 0.6 ^ABb^	37.5 ± 0.4 ^Ab^
	6	45.5 ± 0.6 ^Aa^	41.1 ± 0.5 ^Ab^	39.6 ± 0.3 ^Abc^	38.2 ± 0.3 ^Ac^
a*	1	10.0 ± 0.3 ^Ba^	6.7 ± 0.3 ^Bb^	7.6 ± 0.3 ^Ab^	7.0 ± 0.3 ^Ab^
	3	13.7 ± 0.4 ^Aa^	9.2 ± 0.4 ^Ab^	8.3 ± 0.3 ^Ab^	6.7 ± 0.2 ^Ac^
	6	13.3 ± 0.3 ^Aa^	9.3 ± 0.2 ^Ab^	7.4 ± 0.3 ^Ac^	6.3 ± 0.2 ^Ac^
b*	1	8.1 ± 0.3 ^Aa^	9.2 ± 0.3 ^Aa^	8.9 ± 0.3 ^Aa^	8.5 ± 0.3 ^Aa^
	3	8.5 ± 0.3 ^Ab^	9.8 ± 0.2 ^Aa^	8.5 ± 0.3 ^Ab^	8.4 ± 0.2 ^Ab^
	6	9.1 ± 0.2 ^Aa^	9.6 ± 0.3 ^Aa^	8.8 ± 0.1 ^Aa^	8.7 ± 0.2 ^Aa^
∆E*	1	-	4.7 ± 0.5 ^Bb^	6.7 ± 0.7 ^Aab^	7.2 ± 0.7 ^Ba^
	3	-	6.9 ± 0.4 ^Ab^	8.5 ± 0.4 ^Aab^	9.5 ± 0.4 ^Aa^
	6	-	6.2 ± 0.4 ^Abb^	8.7 ± 0.4 ^Aa^	10.4 ± 0.4 ^Aa^

Means ± SEM. Different uppercase superscripts (^A,B^) in the same column indicate significant differences among the same treatment over storage days. Different lowercase superscripts (^a–c^) in the same row indicate significant differences among different treatments on the same day. SEM: Standard error of the mean. Control: patties without *T. molitor* powder addition; HT50: patties with 5% *T. molitor* powder; HT75: patties with 7.5% *T. molitor* powder; HT100: patties with 10% *T. molitor* powder.

**Table 3:** Kramer Shear Force (KSF) and Thiobarbituric Acid Reactive Substances (TBARS) of patties with different levels of *T. molitor* powder during storage.

Parameter	Day	Treatments			
		Control	HT50	HT75	HT100
KSF (N/g)	1	29.38 ± 0.61 ^Aa^	26.88 ± 0.51 ^Ba^	28.68 ± 0.59 ^Ba^	30.32 ± 0.97 ^Ba^
	6	32.86 ± 0.52 ^Ab^	34.92 ± 1.42 ^Ab^	42.45 ± 1.25 ^Aa^	43.34 ± 0.27 ^Aa^
TBARS (mg MDA/kg)	1	1.63 ± 0.02 ^Aa^	1.32 ± 0.01 ^Bb^	1.05 ± 0.02 ^Bc^	0.92 ± 0.01 ^Bd^
	6	1.63 ± 0.01 ^Aa^	1.51 ± 0.01 ^Ab^	1.44 ± 0.02 ^Ac^	1.45 ± 0.02 ^Abc^

Means ± SEM. Different uppercase superscripts (^A,B^) in the same column indicate significant differences among the same treatment over storage days. Different lowercase superscripts (^a–c^) in the same row indicate significant differences among different treatments on the same day. SEM: Standard error of the mean. Control: patties without *T. molitor* powder addition; HT50: patties with 5% *T. molitor* powder; HT75: patties with 7.5% *T. molitor* powder; HT100: patties with 10% *T. molitor* powder.

## Data Availability

The original contributions presented in this study are included in the article. Further inquiries can be directed to the corresponding authors.

## Abbreviations

The following abbreviations are used in this manuscript:

AMSA	American Meat Science Association
AOAC	Association Of Official Analytical Chemists
ANOVA	Analysis of Variance
APHA	American Public Health Association
CFU	Colony-Forming Unit
CL	Cooking Loss
DR	Diameter Reduction
KSF	Kramer Shear Force
LAB	Lactic Acid Bacteria
MDA	Malondialdehyde
TBARS	Thiobarbituric Acid Reactive Substances
